# Attitudes toward HPV Vaccination among Women Aged 27 to 45

**DOI:** 10.5402/2011/670318

**Published:** 2011-04-07

**Authors:** Thomas W. Weiss, Susan L. Rosenthal, Gregory D. Zimet

**Affiliations:** ^1^Global Health Outcomes, Merck & Co., Inc., P.O. Box 4, WP97-A243, 770 Sumneytown Pike, West Point, PA 19486-0004, USA; ^2^Department of Pediatrics, Columbia University, 3959 Broadway, CHC Central/South 1124, New York, NY 10032, USA; ^3^Department of Pediatrics, Indiana University School of Medicine, West 10th Street, HS 1001, Indianapolis, IN 46202, USA

## Abstract

The purpose of this study was to identify attitudes toward HPV vaccination among US women 27 to 45 years of age. A survey was mailed to 2,750 insured US women to assess perceptions of relevance or irrelevance of the HPV vaccine, the underlying reasons, and, for those reporting relevance, the likelihood of vaccination if it became available. Among the 451 eligible respondents, 304 (67.4%) reported that the HPV vaccine was relevant to them, whereas 143 (31.7%) stated that it was not at all relevant. The most common reasons for relevance were protection from cervical cancer (62.8%), vaginal cancer (58.2%), precancerous cells (55.9%), HPV (55.6%), and genital warts (46.4%). Reasons for irrelevance were most commonly being married (54.0%) or in a monogamous relationship (39.6%). Most respondents reporting relevance of the HPV vaccine were likely (33.4%) or extremely likely (37.7%) to receive the vaccine if approved for their age group.

## 1. Introduction

Human papillomavirus (HPV) is the most common sexually transmitted infection in the United States [1]. Low-risk HPV types 6 and 11 cause 90% of cases of genital warts, while high-risk HPV types 16 and 18 are responsible for 70% of cervical cancers and also cause cancers of the anus, vagina, vulva, and head and neck [2, 3]. As of 2004, the prevalence of HPV infection was 26.8% among US women aged 14–59 [4]. A gradual decline in prevalence with age is apparent in women in their fifties (19.6%) compared to those in their forties (25.2%) and thirties (27.5%) [4]. 

Two HPV vaccines are licensed in the United States. A quadrivalent vaccine targeting HPV types 6, 11, 16, and 18 was approved in 2006 for females aged 9–26 [5], and a bivalent vaccine targeting types 16 and 18 was approved in 2009 for females aged 10–25 [6]. The US Advisory Committee on Immunization Practices (ACIP) recommends routine vaccination of girls aged 11-12 and catch-up vaccination of females aged 13–26 with either vaccine [7]. Both vaccines have been studied in women over 26 [8, 9] and are approved or under regulatory review in several countries for use in older women [10]. 

HPV vaccine acceptability is generally high in women over 26 [11, 12] but a number of studies report a decrease in vaccine acceptability with increasing age [13–18], which raises the questions of whether and why older women think the vaccine is relevant to them. Thus, this study assessed the relevance of HPV vaccination to US women aged 27–45 as well as their intentions regarding HPV vaccination (if approved in the future for this age group).

## 2. Materials and Methods

### 2.1. Study Design

This is the third of several reports on a survey of adult women's attitudes toward the HPV vaccine [19, 20]. Subjects were identified using medical and pharmacy claims from a large US managed care plan affiliated with i3 Innovus. The health plan provides fully insured coverage of physician, hospital, and prescription drug services to more than 15 million patients across the United States. A self-administered survey assessed personal relevance of the HPV vaccine and intentions regarding HPV vaccination (if offered in the future). The Copernicus Group Independent Review Board approved the study design.

### 2.2. Study Sample

Women were identified in the health plan database if they had an outpatient medical claim on an index date occurring between January 1 and April 30, 2007 (the identification period), were 27–45 years of age on the index date, and were continuously enrolled in the health plan during an observation period extending from 6 months before to 12 months following the index date. Women were excluded if they had a medical claim related to cervical cancer during the 18-month observation period or a claim for HPV vaccination during the identification period. A total of 141,130 women met the above criteria. Samples of 1,375 women were randomly selected from among those aged 27–34 and 35–45, for a total of 2,750 study subjects.

### 2.3. Survey Administration

Survey packets were mailed beginning in April 2008. Packets contained an invitation letter, an informed consent form, a 7-page survey, and a payment of $10 as compensation for participation. the reminder postcards were sent one week following the initial mailing, and a second survey packet was sent to nonrespondents after three weeks. The total survey collection period was 8 weeks.

### 2.4. Survey Content

The survey collected information on demographic characteristics (age, ethnicity, educational history, marital status, and employment status), overall health status, and general attitudes about reproductive health. A series of questions then assessed the respondents' attitudes toward HPV vaccination. After being asked to read a short paragraph about HPV, the HPV vaccine, and the types of diseases the vaccine prevents, respondents were asked “How relevant is this vaccine to you?” Possible answers were “extremely relevant,” “very relevant,” “relevant,” “slightly relevant,” and “not at all relevant.” 

If the respondent indicated that the vaccine was relevant (defined as answers ranging from extremely to slightly relevant), she was asked about her reasons for believing so. Possible reasons included the desire for protection from HPV and its various sequelae and beliefs about the safety and efficacy of the vaccine. Respondents who thought the vaccine was relevant to them were asked with which type of health care provider they would specifically discuss the vaccine. Possible answers were “gynecologist (OB/GYN),” “general/family practitioner (GP/FP),” “internist or internal medicine doctor (IM),” and “nurse practitioner/physician's assistant.” They were also asked how likely they would be to get the vaccine if it were offered in the future: “extremely likely,” “likely,” “unsure,” “unlikely,” or “extremely unlikely.” 

Respondents who indicated that the vaccine was not relevant to them were asked why they believed it was not relevant. Possible answers included not being at risk for HPV and its sequelae, needing more information about the vaccine, and being married or in a monogamous relationship.

### 2.5. Data Analysis

A descriptive analysis was conducted on the entire study sample. The results are presented as the number and percentage of respondents who gave a particular answer to each survey question. Demographic characteristics are given for all respondents, but the frequencies of reasons given for relevance or irrelevance of the vaccine are presented as a proportion of respondents in those subgroups. A stratified analysis of age groups 27–34 and 35–45 was carried out to determine whether there were any attitudinal differences between them.

## 3. Results

### 3.1. Respondent Characteristics

Of 2,750 surveys sent out, 457 were completed and returned, for an overall response rate of 16.6%. Six respondents were subsequently found to be ineligible due to HPV vaccination or disenrollment from the health plan, leaving 451 surveys available for analysis. Respondents (Table 1) were predominantly white (84.0%), married (75.6%), and employed fulltime (67.8%). Their mean (SD) age was 36.8 (5.4), and most (88.2%) had heard of an HPV or cervical cancer vaccine prior to receiving the survey packet. Response rates were 21.4% for 35–45-year olds and 11.4% for 27–34-year olds, so that the majority of the study subjects (65.2%) were in the older age group (Table 1).

### 3.2. Attitudes toward HPV Vaccination among Respondents for Whom the Vaccine Was Relevant

Of the 451 respondents, 304 (67.4%) reported that the HPV vaccine was relevant to them. Among these women, the most frequent reasons given for believing the vaccine to be relevant (Table 2) were protection from cervical cancer (62.8%), vaginal cancer (58.2%), precancerous cells (55.9%), and HPV (55.6%). Concern about public health was cited by 47.0% of women and protection against genital warts by 46.4%. A stratified analysis of 27–34 and 35–45-year olds found no statistically significant differences in their perceptions of relevance of the vaccine.

**Figure 1:**
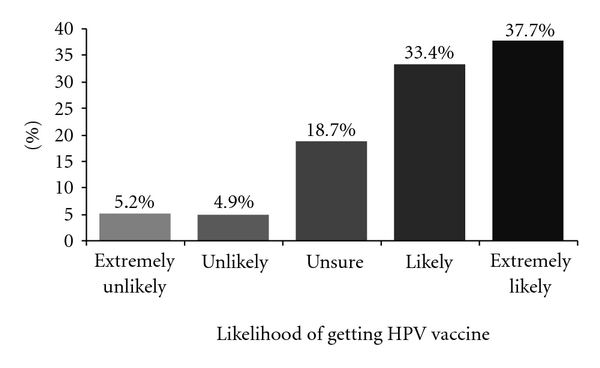
Likelihood of getting the HPV vaccine if offered in the future (among respondents for whom the vaccine was relevant (*N* = 304)).

Almost all (92.8%) women who thought the HPV vaccine relevant would plan to discuss the vaccine with a gynecologist (Table 3). Among women who found vaccination relevant, 71.1% were likely or extremely likely to be vaccinated if the HPV vaccine were offered in the future to women in their age group (Figure 1), while 10.1% were unlikely or extremely unlikely to be vaccinated.

### 3.3. Attitudes toward HPV Vaccination among Respondents for Whom the Vaccine Was Not Relevant

There were 143 (31.7%) respondents who felt the HPV vaccine was not relevant to them. Among this group, the most frequently reported reasons for HPV vaccination not being relevant (Table 4) were being married (54.0%), being in a monogamous relationship (39.6%), not being at risk for HPV (25.2%) or genital warts (19.4%), and concerns about vaccine safety (19.4%) and effectiveness (17.3%).

## 4. Discussion

In this insured population of women aged 27 to 45, about two-thirds felt the HPV vaccine was relevant to them, and, among these, nearly three in four were likely or extremely likely to get vaccinated if HPV vaccination were offered in the future to women of their age. One-third saw the vaccine as not relevant, most commonly because they were married or in a monogamous relationship or because they believed that they were at low risk for getting HPV infection and HPV-related disease.

Only one other survey has specifically elicited the reasons older women have for wanting or not wanting the HPV vaccine [13, 21]. In the first of two reports on that survey, the most frequent reasons for wanting the HPV vaccine were a desire to stay healthy (48.9%), prevention of cervical cancer (42.8%), and prevention of genital warts (33.5%) [21]. The most common reasons for not wanting the vaccine were being in a monogamous relationship (29.5%) and perception of low risk of HPV infection (15.0%). In the second and more quantitative report, women in a monogamous relationship had half the odds (OR 0.46) of wanting the HPV vaccine, while women who felt at risk for HPV infection had higher odds of wanting the vaccine (OR 2.14) [13].

Younger women have similar reasons for wanting or not wanting the HPV vaccine. Belief in the severity of cervical cancer was associated with higher intent to be vaccinated among 13–26-year olds [22], and knowledge of HPV and its relationship to cervical cancer has been found to correlate with vaccine acceptability in young adult women [14, 23]. Similarly, among nonvaccinees in this age range, a frequent reason for inaction regarding HPV vaccination is being married or in a monogamous relationship [19]. Other reasons for inaction or lack of interest are also common and include a perceived lack of need, usually due to sexual inactivity or low risk, and concerns about the safety of the vaccine [24, 25]. 

In previous studies of nonvaccinees, 7 to 14.6% of women aged 18–26 cited cost as a reason for not being vaccinated [19, 25]. Cost was also a barrier reported by college women who had chosen not to receive the vaccine [26]. However, in this survey of women over 26, fewer than 1.0% of the total number of respondents expressed concerns regarding affordability. This was perhaps because they all had insurance coverage—a similar study of a nationally representative sample of US women aged 27–55 found that cost was the most influential factor in HPV vaccine acceptability across multiple sociodemographic and health variables [12].

Awareness of HPV and the HPV vaccine is generally high in women over 26. The National Immunization Survey of 2007 found that 82.9% of US women aged 27–48 had heard of HPV and 76.5% had heard of the HPV vaccine (versus 88.6% and 82.8%, resp., of women aged 18–26) [25]. In Canada, 84.7% of adult women (mean age 33) surveyed in 2007 indicated that they had heard of HPV, though only 39.8% had heard of the HPV vaccine [15]. Most respondents in this study (88.2%) had heard of an HPV or cervical cancer vaccine prior to receiving the survey packet. Although HPV awareness was lower (<60%) in 2005-2006 in some rural regions of the United States [18, 27], it can be expected to increase over time as HPV vaccination becomes more common.

Health care providers are women's most trusted source of information about HPV and the HPV vaccine [28, 29]. In fact, physician discussion and recommendation are important predictors of vaccination in younger women [20, 25, 29, 30]. One recent survey suggests that physicians may also be influential for women aged 27–45: among women in this age group who were not aware of the vaccine or who had not received a physician's recommendation, 80% reported that they would get vaccinated if a health care provider recommended it [25]. 

Physician recommendation may play an especially important role for married women, who in both younger and older adult age groups feel less at risk for HPV infection. Their perception is supported by prevalence data showing that HPV infection is twice as likely in never married women and three times as likely in widowed/divorced/separated or cohabiting women compared to married women [4]. Nevertheless, HPV infection is still prevalent (17.3%) among married women [4], and our previous analysis showed physician recommendation to be a stronger predictor of vaccination than marital status [20].

Because the prevalence of HPV peaks among women aged 20–24 [4], one question of particular relevance to older women is whether vaccination can prevent HPV-related disease in previously exposed or currently infected individuals. Clinical studies have shown that, in women infected with some, but not all, of the four HPV types targeted by the quadrivalent vaccine, the vaccine prevented disease caused by the remaining HPV types [31]. Preliminary findings are similar for the bivalent vaccine [32]. Another question is whether older women are at risk for acquiring new HPV infections. Studies conducted in several countries indicate that, while incidence of HPV infection declines in the fourth decade compared to earlier ages, there remains a significant risk of HPV infection in sexually active women of all ages [32]. In Canada, for instance, the one-year acquisition rate of oncogenic HPV infection was 9.7% among women aged 25–49 [33]. Furthermore, a large cohort study in Costa Rica found that type-specific persistence increases with age, particularly for HPV-16 [34]. 

One limitation of our study was the low survey response rate. We were able to compare the age and geographic locations of respondents and nonrespondents using claims data. There were no statistically significant differences between these two groups in age, whether analyzed as a continuous variable or as a categorical variable (age categories 27 to 35 and 36 to 45 years). Similar proportions of respondents and nonrespondents resided in the Northeast and West, but respondents were more likely than nonrespondents to be located in the Midwest (32.6% versus 24.8%, *P* < .001) and less likely to reside in the South (46.1% versus 55.6%, *P* < .001). It is possible that this geographic imbalance may have produced bias in the estimation of reports of HPV vaccine relevance. In addition, respondents were given limited information on the efficacy of the vaccine, which may have affected their perceptions of relevance. Because of the study population chosen, the results may not be generalizable to women living outside the United States or women without health insurance. Finally, intention to get vaccinated may not translate into actual uptake [35], so the interpretation of the results pertaining to future action is limited.

## 5. Conclusions

In this sample of women aged 27 to 45, most felt the HPV vaccine was relevant to them because of its ability to protect them from HPV infection and HPV-related diseases. The majority of women reporting relevance were likely to consider vaccination if the vaccine became available to their age group. Women who did not feel the vaccine was relevant did not perceive themselves at risk for HPV infection or HPV-related diseases.

## Figures and Tables

**Table 1:** Respondent characteristics.

	*N*	%
Total^a^	451	100
Ages 27–34	157	34.8
Ages 35–45	294	65.2

Marital status, *N* = 451		
Married	341	75.6
Single, never married	59	13.1
Divorced	41	9.1
Separated/widowed	10	2.4

Overall health status, *N* = 443		
Poor	3	0.7
Fair	32	7.2
Good	131	29.6
Very good	203	45.8
Excellent	74	16.7

Education, *N* = 450		
Some high school or less	4	0.9
High school or equivalent (e.g., GED)	38	8.4
Some college, but no degree	94	20.9
Two-year degree or college graduate	227	50.4
Graduate school	87	19.3

Race/ethnicity (check all that apply), *N* = 451		
White	378	83.8
Hispanic or Latino	30	6.7
Black/African-American	29	6.4
Other^b^	105	23.3

Employment status (check all that apply),		
Fulltime	306	67.8
Parttime	48	10.6
Homemaker/not employed	78	17.3
Student	12	2.7

^a^Missing responses are indicated by the Ns for each response category.

^b^American Indian, Alaska Native, Asian, Native Hawaiian, Pacific Islander, and Other.

**Table 2:** Reasons for relevance of HPV vaccination.^a^

	*N*	%
I want to be protected from cervical cancer	191	62.8
I want to be protected from vaginal cancer	177	58.2
I want to be protected from precancerous cells	170	55.9
I want to be protected from getting HPV	169	55.6
It is the right thing to do for public health (stop the spread of HPV)	143	47.0
I want to be protected from genital warts	141	46.4
It is better to have the vaccine than to not have it	127	41.8
This vaccine is safe	82	27.0
This vaccine is effective	75	24.7
I am not in a monogamous relationship	11	3.6
Other$\;\hat{\;}$	82	27.0

^a^Total *N* = 304. Relevance was defined as responses of slightly relevant, relevant, very relevant, or extremely relevant. Respondents were instructed to “check all that apply.”

$\hat{\;}\;$Examples include having personal or family history of HPV infection or cervical disease or having one or more daughters to protect against HPV-related disease.

**Table 3:** Types of health care providers with whom respondents would discuss the HPV vaccine.^a^

	*N*	%
Gynecologist (OB/GYN)	282	92.8
General/family practitioner (GP/FP)	105	34.5
Nurse practitioner/physician's assistant	49	16.1
Internist or internal medicine doctor (IM)	28	9.2
Other$\;\hat{\;}$	14	4.6

^a^Among respondents who considered the HPV vaccine relevant. Total *N* = 304. Respondents were instructed to “check all that apply”.

$\hat{\;}\;$Example: daughter's pediatrician.

**Table 4:** Reasons why HPV vaccination is not relevant.^a^

	*N*	%
I am married	75	54.0
I am in a monogamous relationship (one partner at a time)	55	39.6
I am not at risk for getting HPV	35	25.2
I am not at risk for getting genital warts	27	19.4
I am not convinced/need more information on the vaccine's safety	27	19.4
I am not convinced/need more information on the vaccine's effectiveness	24	17.3
I am not at risk for getting cervical cancer	14	10.1
I am not at risk for getting vaginal cancer	7	5.0
I do not currently have sexual relations	7	5.0
I cannot afford this vaccine	2	1.4
Other$\;\hat{\;}$	62	44.6

^a^Four of the 143 respondents who reported that the vaccine was not at all relevant did not answer this question, leaving 139 total responses from which to calculate the percentages shown here. Respondents were instructed to “check all that apply.”

$\hat{\;}\;$Examples include outside of indicated age range (i.e., >age 26), having personal history of HPV infection or cervical disease.

## References

[1] Weinstock H, Berman S, Cates W (2004). Sexually transmitted diseases among American youth: incidence and prevalence estimates, 2000. *Perspectives on Sexual and Reproductive Health*.

[2] Huang CM (2008). Human papillomavirus and vaccination. *Mayo Clinic Proceedings*.

[3] Castellsagué X (2008). Natural history and epidemiology of HPV infection and cervical cancer. *Gynecologic Oncology*.

[4] Dunne EF, Unger ER, Sternberg M (2007). Prevalence of HPV infection among females in the United States. *Journal of the American Medical Association*.

[5] Food and Drug Administration Gardasil Approval Letter. http://www.fda.gov/BiologicsBloodVaccines/Vaccines/ApprovedProducts/default.htm.

[6] Food and Drug Administration Cervarix Approval Letter. http://www.fda.gov/BiologicsBloodVaccines/Vaccines/ApprovedProducts/default.htm.

[7] (2010). FDA licensure of bivalent human papillomavirus vaccine (HPV2, Cervarix) for use in females and updated HPV vaccination recommendations from the Advisory Committee on Immunization Practices (ACIP). *Morbidity and Mortality Weekly Report*.

[8] Muñoz N, Manalastas R, Pitisuttithum P (2009). Safety, immunogenicity, and efficacy of quadrivalent human papillomavirus (types 6, 11, 16, 18) recombinant vaccine in women aged 24-45 years: a randomised, double-blind trial. *The Lancet*.

[9] Schwarz TF, Spaczynski M, Schneider A (2009). Immunogenicity and tolerability of an HPV-16/18 AS04-adjuvanted prophylactic cervical cancer vaccine in women aged 15–55 years. *Vaccine*.

[10] Bornstein J (2009). The HPV vaccines—which to prefer?. *Obstetrical and Gynecological Survey*.

[11] Black LL, Zimet GD, Short MB, Sturm L, Rosenthal SL (2009). Literature review of human papillomavirus vaccine acceptability among women over 26 years. *Vaccine*.

[12] Stupiansky NW, Rosenthal SL, Wiehe SE, Zimet GD (2010). Human papillomavirus vaccine acceptability among a national sample of adult women in the USA. *Sexual Health*.

[13] Ferris DG, Waller JL, Owen A, Smith J (2008). HPV vaccine acceptance among mid-adult women. *Journal of the American Board of Family Medicine*.

[14] Jones M, Cook R (2008). Intent to receive an HPV vaccine among university men and women and implications for vaccine administration. *Journal of American College Health*.

[15] Lenehan JG, Leonard KC, Nandra S, Isaacs CR, Mathew A, Fisher WA (2008). Women’s knowledge, attitudes, and intentions concerning Human Papillomavirus vaccination: findings of a waiting room survey of obstetrics-gynaecology outpatients. *Journal of Obstetrics and Gynaecology Canada*.

[16] Sperber NR, Brewer NT, Smith JS (2008). Influence of parent characteristics and disease outcome framing on HPV vaccine acceptability among rural, Southern women. *Cancer Causes and Control*.

[17] Sauvageau C, Duval B, Gilca V, Lavoie F, Ouakki M (2007). Human Papilloma Virus vaccine and cervical cancer screening acceptability among adults in Quebec, Canada. *BMC Public Health*.

[18] Hopenhayn C, Christian A, Christian WJ, Schoenberg NE (2007). Human papillomavirus vaccine: knowledge and attitudes in two Appalachian Kentucky counties. *Cancer Causes and Control*.

[19] Zimet GD, Weiss TW, Rosenthal SL, Good MB, Vichnin MD (2010). Reasons for non-vaccination against HPV and future vaccination intentions among 19–26 year-old women. *BMC Women's Health*.

[20] Rosenthal SL, Weiss TW, Zimet GD, Ma L, Good MB, Vichnin MD (2011). Predictors of HPV vaccine uptake among women aged 19–26: importance of a physician’s recommendation. *Vaccine*.

[21] Ferris DG, Waller JL, Owen A, Smith J (2007). Midadult women's attitudes about receiving the prophylactic human papillomavirus vaccine. *Journal of Lower Genital Tract Disease*.

[22] Kahn JA, Rosenthal SL, Jin Y, Huang B, Namakydoust A, Zimet GD (2008). Rates of human papillomavirus vaccination, attitudes about vaccination, and human papillomavirus prevalence in young women. *Obstetrics and Gynecology*.

[23] Kahn JA, Rosenthal SL, Hamannn T, Bernstein DI (2003). Attitudes about human papillomavirus vaccine in young women. *International Journal of STD and AIDS*.

[24] Grant D, Kravitz-Wirtz N, Breen N, Tiro JA, Tsui J (2009). One in four California adolescent girls have had human papillomavirus vaccination. *Policy Brief (UCLA Center for Health Policy Research)*.

[25] Jain N, Euler GL, Shefer A, Lu P, Yankey D, Markowitz L (2009). Human papillomavirus (HPV) awareness and vaccination initiation among women in the United States, National Immunization Survey-Adult 2007. *Preventive Medicine*.

[26] Allen JD, Mohllajee AP, Shelton RC, Othus MKD, Fontenot HB, Hanna R (2009). Stage of adoption of the human papillomavirus vaccine among college women. *Preventive Medicine*.

[27] Cates JR, Brewer NT, Fazekas KI, Mitchell CE, Smith JS (2009). Racial differences in HPV knowledge, HPV vaccine acceptability, and related beliefs among rural, Southern women. *Journal of Rural Health*.

[28] Friedman AL, Shepeard H (2007). Exploring the knowledge, attitudes, beliefs, and communication preferences of the general public regarding HPV: findings from CDC Focus Group Research and implications for practice. *Health Education and Behavior*.

[29] Caskey R, Lindau ST, Alexander GC (2009). Knowledge and early adoption of the HPV vaccine among girls and young women: results of a national survey. *Journal of Adolescent Health*.

[30] Reiter PL, Brewer NT, Gottlieb SL, McRee AL, Smith JS (2009). Parents’ health beliefs and HPV vaccination of their adolescent daughters. *Social Science and Medicine*.

[31] Markowitz LE, Dunne EF, Saraiya M, Lawson HW, Chesson H, Unger ER (2007). Quadrivalent Human Papillomavirus Vaccine: recommendations of the Advisory Committee on Immunization Practices (ACIP). *Morbidity and Mortality Weekly Report*.

[32] Castellsagué X, Schneider A, Kaufmann AM, Bosch FX (2009). HPV vaccination against cervical cancer in women above 25 years of age: key considerations and current perspectives. *Gynecologic Oncology*.

[33] Sellors JW, Karwalajtys TL, Kaczorowski J (2003). Incidence, clearance and predictors of human papillomavirus infection in women. *Canadian Medical Association Journal*.

[34] Castle PE, Schiffman M, Herrero R (2005). A prospective study of age trends in cervical human papillomavirus acquisition and persistence in Guanacaste, Costa Rica. *Journal of Infectious Diseases*.

[35] Conroy K, Rosenthal SL, Zimet GD (2009). Human papillomavirus vaccine uptake, predictors of vaccination, and self-reported barriers to vaccination. *Journal of Women’s Health*.

